# Supplementary material for: Partitioning Entropy with Action Mechanics: Predicting Chemical Reaction Rates and Gaseous Equilibria of Reactions of Hydrogen from Molecular Properties
Source: Entropy (Basel). 2021 Aug 16;23(8):1056. doi: 10.3390/e23081056 (PMC8392617; doi:10.3390/e23081056)
Supplement: Supplementary file 1 [file entropy-23-01056-s001.zip › entropy-1316017-supplementary.pdf]

```
ENTROPY6/CAL Sep 17, 2014
Uses action @ to calculate entropy S
For diatomics and linear triatomics,
input mass of terminal atioms in L & N
and central atom or monatomic in M
For other polyatomics, input
Ia, Ib, and Ic as principal moments
of inertia instead.
Input electronic multiplicity as Q
Get vibrational entropy using eqn
Sv=Rx/(e^x-1) - Rln(1-e^-x);x=hv/kT
Get vibrational kinetic heat using
Cvv=Rx^2/2(coshx-1) ex Moore
Input rotational symmetry Z in r99
0560    096    LABEL
0561    090    Z
0562    107    STO MEM
0563    057      9
0564    057      9
0565    119    print
0566    002    HALT
0567    048      0
Input electron multiplicity Qe in r89
0608    096    LABEL
0609    081    Q
0610    107    STO MEM
0611    056      8
0612    057      9
0613    119    print
0614    002    HALT
0615    048      0
Input gas pressure P (atm) in r01
0656    096    LABEL
0657    080    P
0658    107    STO MEM
0659    048      0
0660    049      1
0661    119    print
0662    002    HALT
0663    048      0
Input diatomic mass1 L in r02
0704    096    LABEL
0705    076    L
0706    107    STO MEM
0707    048      0
0708    050      2
0709    119    print
0710    002    HALT
0711    048      0
0712    096    LABEL
0713    077    M
Input monatomic mass M in r03
0754    107    STO MEM
0755    048      0
0756    051      3
0757    119    print
0758    002    HALT
0759    048      0
Input diatomic mass 2 N in r04
0800    096    LABEL
0801    078    N
0802    107    STO MEM
0803    048      0
0804    052      4
0805    119    print
0806    002    HALT
0807    048      0
Input atomic mass3 O in r05
0848    096    LABEL
0849    079    O
0850    107    STO MEM
0851    048      0
0852    053      5
0853    119    print
0854    002    HALT
0855    048      0
Input atomic mass4 J in r06
0896    096    LABEL
0897    074    J
0898    107    STO MEM
0899    048      0
0900    054      6
0901    119    print
0902    002    HALT
0903    048      0
Input atomic mass5 Y in r07
0944    096    LABEL
0945    089    Y
0946    107    STO MEM
0947    048      0
0948    055      7
0949    119    print
0950    002    HALT
0951    048      0
0952    119    print
0953    002    HALT
0954    048      0
Input bond length r1=R in r09
0995    096    LABEL
0996    082    R
0997    107    STO MEM
0998    048      0
0999    057      9
1000    119    print
1001    002    HALT
1002    048      0
Input bond length r2=S in r10
1043    096    LABEL
1044    083    S
1045    107    STO MEM
1046    049      1
1047    048      0
1048    119    print
1049    002    HALT
1050    048      0
Input bond length r3=T in r11
1091    096    LABEL
1092    084    T
```

```
1093    107    STO MEM
1094    049      1
1095    049      1
1096    119    print
1097    002    HALT
1098    048      0
Input IrA rot. moment inertia in r12
1139    096    LABEL
1140    065    A
1141    107    STO MEM
1142    049      1
1143    050      2
1144    119    print
1145.   002    HALT
1146    048      0
Input IrB rot. moment inertia in r13
1187    096    LABEL
1188    066    B
1189    107    STO MEM
1190    049      1
1191    051      3
1192    119    print
1193    002    HALT
1194    048      0
Input IrC rot. moment inertia in r14
1235    096    LABEL
1236    067    C
1237    107    STO MEM
1238    049      1
1239    052      4
1240    119    print
1241    002    HALT
1242    109    RCL MEM
Input frequency 1 w=c/v, F
1283    096    LABEL
1284    070    F
1285    107    STO MEM
1286    051      3
1287    049      1
1288    119    print
1289    002    HALT
1290    048      0
Input frequency 2, G
1331    096    LABEL
1332    071    G
1333    107    STO MEM
1334    051      3
1335    050      2
1336    119    print
1337    002    HALT
1338    048      0
Input frequency 3, H
1379    096    LABEL
1380    072    H
1381    107    STO MEM
1382    051      3
1383    051      3
1384    119    print
1385    002    HALT
1386    048      0
Input frequency 4, I
1427    096    LABEL
1428    073    I
1429    107    STO MEM
1430    051      3
1431    052      4
1432    119    print
1433    002    HALT
1434    048      0
Input degeneracy F, D
1475    096    LABEL
1476    068    D
1477    107    STO MEM
1478    051      3
1479    053      5
1480    119    print
1481    002    HALT
1482    048      0
Input degeneracy G, E
1523    096    LABEL
1524    069    E
1525    107    STO MEM
1526    051      3
1527    054      6
1528    119    print
1529    002    HALT
1530    048      0
Input degeneracy W, for H and I
1571    096    LABEL
1572    087    W
1573    107    STO MEM
1574    051      3
1575    055      7
1576    119    print
1577    002    HALT
1578    048      0
Input degrees Kelvin K in r08
1619    096    LABEL
1620    075    K
1621    107    STO MEM
1622    048      0
1623    056      8
1624    119    print
1625    002    HALT
Execute by ShiftX
1666    096    LABEL
1667    088    X
Store H atomic mass in g in r95
1708    049      1
1709    046      .
1710    054      6
1711    055      7
1712    051      3
1713    053      5
1714    050      2
1715    053      5
1716    101      EE
1717    050      2
1718    052      4
1719    111      +/-
```

```
1720    107    STO MEM
1721    057      9
1722    053      5
Store gas constant R in r96
1763    056      8
1764    046      .
1765    051      3
1766    049      1
1767    052      4
1768    052      4
1769    054      6
1770    050      2
1771    049      1
1772    055      7
1773    053      5
1774    107    STO MEM
1775    057      9
1776    054      6
Store Boltzmann's constant k in r97
1817    049      1
1818    046      .
1819    051      3
1820    056      8
1821    048      0
1822    054      6
1823    052      4
1824    056      8
1825    056      8
1826    101      EE
1827    049      1
1828    054      6
1829    111      +/-
1830    107    STO MEM
1831    057      9
1832    055      7
Store Planck's red. quantum h- in r98
1873    049      1
1874    046      .
1875    048      0
1876    053      5
1877    052      4
1878    057      9
1879    049      1
1880    056      8
1881    053      5
1882    101      EE
1883    050      2
1884    055      7
1885    111      +/-
1886    107    STO MEM
1887    057      9
1888    056      8
1889    050      2
1890    046      .
1891    057      9
1892    057      9
1893    055      7
1894    057      9
1895    050      2
1896    052      4
1897    053      5
1898    056      8
1899    101      EE
1900    049      1
1901    048      0
1902    119    print
Store c in r94
1943    107    STO MEM
1944    057      9
1945    052      4
1946    040      (
1947    040      (
Recall pressure (atm), find radius rt
Recall T/P/298.15
2028    109    RCL MEM
2029    048      0
2030    056      8
2031    047      /
2032    109    RCL MEM
2033    048      0
2034    049      1
2035    047      /
2036    050      2
2037    057      9
2038    056      8
2039    046      .
2040    049      1
2041    053      5
2042    061      =
2043    119    print
2044    092    Root
2045    051      3
2046    042      x
2047    049      1
2048    046      .
2049    055      7
2050    049      1
2051    056      8
2052    056      8
2053    041      )
2054    042      x
2055    049      1
2056    101      EE
2057    055      7
2058    111      +/-
2059    061      =
2060    119    print
2061    113    Square
2062    041      )
2063    042      x
2064    040      (
Recall AW 1 from r02
2105    109    RCL MEM
2106    048      0
2107    050      2
2108    043      +
Recall AW 2 from r03
2149    109    RCL MEM
2150    048      0
2151    051      3
```

```
2152    043      +
Recall  AW 3 from r04
2193    109     RCL MEM
2194    048      0
2195    052      4
2196    043      +
Recall  AW 4 from r05
2237    109     RCL MEM
2238    048      0
2239    053      5
2240    043      +
Recall  AW 5 from r06
2281    109     RCL MEM
2282    048      0
2283    054      6
Recall  AW 6 from r07
2324    109     RCL MEM
2325    048      0
2326    055      7
2327    041      )
2328    042      x
2329    109     RCL MEM
2330    057      9
2331    053      5
2332    061      =
Print  and  store It in r15
2373    119     print
2374    107     STO MEM
2375    049      1
2376    053      5
2377    042      x
Recall  k
2418    109     RCL MEM
2419    057      9
2420    055      7
2421    042      x
2422    051      3
2423    042      x
2424    109     RCL MEM
2425    048      0
2426    056      8
2427    061      =
2428    092     Root
2429    050      2
2430    061      =
Print  and  store action ∂t in r16
2471    119     print
2472    107     STO MEM
2473    049      1
2474    054      6
2475    047      /
Recall  h-
2516    109     RCL MEM
2517    057      9
2518    056      8
2519    061      =
Print  and  store ∂/h- in r17
2560    107     STO MEM
2561    049      1
2562    055      7
2563    119     print
2564    109     RCL MEM
2565    049      1
2566    055      7
2567    121     Cube
2568    061      =
2569    047      /
2570    049      1
2571    048      0
2572    046      .
2573    050      2
2574    050      2
2575    057      9
2576    055      7
2577    061      =
2578    110     LOG e
2579    042      x
2580    109     RCL MEM
2581    057      9
2582    054      6
2583    061      =
2584    107     STO MEM
2585    049      1
2586    056      8
2587    109     RCL MEM
2588    049      1
2589    056      8
2590    119     print
2591    107     STO MEM
2592    054      6
2593    049      1
2594    109     RCL MEM
2595    054      6
2596    049      1
Print  cumulative -G/T
2637    119     print
2638    109     RCL MEM
2639    057      9
2640    054      6
2641    042      x
2642    050      2
2643    046      .
2644    053      5
2645    061      =
2646    117     SUM MEM
2647    049      1
2648    056      8
2649    107     STO MEM
2650    054      6
2651    050      2
2652    109     RCL MEM
2653    054      6
2654    050      2
Print  cmulative H/T
2695    119     print
2696    109     RCL MEM
2697    049      1
2698    056      8
2699    119     print
Print  St with Qe=1
```

```
Check for Qe eg O2 3, N2O 4
2780    040     (
2781    049     1
2782    045     -
2783    109     RCL MEM
2784    056     8
2785    057     9
2786    041     )
2787    038     If zero
2788    104     Hyperbol
2789    109     RCL MEM
2790    056     8
2791    057     9
2792    110     LOG e
2793    042     x
2794    109     RCL MEM
2795    057     9
2796    054     6
2797    061     =
2798    117     SUM MEM
2799    054     6
2800    049     1
2801    117     SUM MEM
2802    049     1
2803    056     8
2804    109     RCL MEM
2805    049     1
2806    056     8
Print St with Qe>1
2847    119     print
2848    109     RCL MEM
2849    054     6
2850    049     1
Print cumulative -G/T
2891    119     print
2892    096     LABEL
2893    104     Hyperbol
Test if monatomic
2934    109     RCL MEM
2935    048     0
2936    052     4
2937    038     If zero
2938    115     Sine
Calculate rotational entropy
Test if non-linear
3019    040     (
3020    048     0
3021    045     -
Recall Ia
3062    109     RCL MEM
3063    049     1
3064    050     2
3065    041     )
3066    064     Inverse
3067    037     If pos
3068    116     Tangent
3069    040     (
Calculate reduced mass
3110    040     (
3111    109     RCL MEM
3112    048     0
3113    050     2
3114    042     x
3115    109     RCL MEM
3116    048     0
3117    052     4
3118    041     )
3119    047     /
3120    040     (
3121    109     RCL MEM
3122    048     0
3123    050     2
3124    043     +
3125    109     RCL MEM
3126    048     0
3127    052     4
3128    041     )
3129    042     x
Recall H atom mass
3170    109     RCL MEM
3171    057     9
3172    053     5
3173    042     x
3174    040     (
3175    109     RCL MEM
3176    048     0
3177    057     9
3178    043     +
3179    109     RCL MEM
3180    049     1
3181    048     0
3182    041     )
3183    113     Square
3184    041     )
Print and store Ir in r19
3225    119     print
3226    107     STO MEM
3227    049     1
3228    057     9
3229    040     (
3230    109     RCL MEM
3231    049     1
3232    057     9
3233    042     x
Recall degrees K
3274    109     RCL MEM
3275    048     0
3276    056     8
3277    042     x
Recall k
3318    109     RCL MEM
3319    057     9
3320    055     7
3321    042     x
3322    050     2
3323    041     )
3324    061     =
3325    092     Root
3326    050     2
3327    061     =
```

```
Print and store ∂r in r20
3368    119    print
3369    107    STO MEM
3370    050      2
3371    048      0
Calculate Sr
3412    040      (
3413    109    RCL MEM
3414    050      2
3415    048      0
3416    047      /
Recall h-
3457    109    RCL MEM
3458    057      9
3459    056      8
3460    041      )
Print and store ∂/h- in r21
3501    119    print
3502    107    STO MEM
3503    050      2
3504    049      1
3505    113    Square
3506    047      /
Insert symmetry factor
3547    109    RCL MEM
3548    057      9
3549    057      9
3550    061      =
3551    110    LOG e
3552    042      x
Recall R
3593    109    RCL MEM
3594    057      9
3595    054      6
3596    061      =
Print and store -G/T in r22
3637    119    print
3638    107    STO MEM
3639    050      2
3640    050      2
3641    117    SUM MEM
3642    054      6
3643    049      1
3644    109    RCL MEM
3645    054      6
3646    049      1
3647    119    print
3648    109    RCL MEM
3649    057      9
3650    054      6
3651    117    SUM MEM
3652    054      6
3653    050      2
3654    117    SUM MEM
3655    050      2
3656    050      2
3657    109    RCL MEM
3658    054      6
3659    050      2
Print cumulative C
3700    119    print
3701    109    RCL MEM
3702    050      2
3703    050      2
3704    119    print
3705    043      +
3706    109    RCL MEM
3707    049      1
3708    056      8
3709    061      =
Print St+Sr
3750    119    print
3751    034    Set flag
3752    049      1
3753    034    Set flag
3754    053      5
3755    040      (
3756    048      0
3757    045      -
3758    109    RCL MEM
3759    051      3
3760    049      1
3761    041      )
3762    064    Inverse
3763    037    If pos
3764    086    V
3765    109    RCL MEM
3766    050      2
3767    050      2
3768    042      x
3769    109    RCL MEM
3770    048      0
3771    056      8
3772    061      =
Print cumulative ST
3813    119    print
3814    109    RCL MEM
3815    054      6
3816    049      1
3817    042      x
3818    109    RCL MEM
3819    048      0
3820    056      8
3821    061      =
Print cumulative -G
3862    119    print
3863    109    RCL MEM
3864    054      6
3865    050      2
3866    042      x
3867    109    RCL MEM
3868    048      0
3869    056      8
3870    061      =
Print cumulative H
3911    119    print
3912    002    HALT
Calculate Sr for polyatomic gases
Sr=Rln[pi^1/2e^3/2∂a∂b∂c/h-^3/z)
Calculate ∂rA
```

```
4033     096     LABEL
4034     116     Tangent
4035     040       (
4036     109     RCL MEM
4037     049       1
4038     050       2
4039     042       x
4040     109     RCL MEM
4041     048       0
4042     056       8
4043     042       x
4044     050       2
4045     042       x
Recall k
4086     109     RCL MEM
4087     057       9
4088     055       7
4089     041       )
4090     092     Root
4091     050       2
4092     061       =
Print and store ∂rA in r23
4133     119     print
4134     107     STO MEM
4135     050       2
4136     051       3
4137     109     RCL MEM
4138     050       2
4139     051       3
4140     047       /
Recall h-
4181     109     RCL MEM
4182     057       9
4183     056       8
4184     061       =
Print and store ∂rA/h- in r24
4225     119     print
4226     107     STO MEM
4227     050       2
4228     052       4
Calculate ∂rB
4269     040       (
4270     109     RCL MEM
4271     049       1
4272     051       3
4273     042       x
4274     109     RCL MEM
4275     048       0
4276     056       8
4277     042       x
4278     050       2
4279     042       x
Recall k
4320     109     RCL MEM
4321     057       9
4322     055       7
4323     041       )
4324     092     Root
4325     050       2
4326     061       =
Print and store ∂rB in r25
4367     119     print
4368     107     STO MEM
4369     050       2
4370     053       5
4371     109     RCL MEM
4372     050       2
4373     053       5
4374     047       /
Recall h-
4415     109     RCL MEM
4416     057       9
4417     056       8
4418     061       =
Print and store ∂rB/h- in r26
4459     119     print
4460     107     STO MEM
4461     050       2
4462     054       6
Calculate ∂rC
4503     040       (
4504     109     RCL MEM
4505     049       1
4506     052       4
4507     042       x
4508     109     RCL MEM
4509     048       0
4510     056       8
4511     042       x
4512     050       2
4513     042       x
Recall k
4554     109     RCL MEM
4555     057       9
4556     055       7
4557     041       )
4558     092     Root
4559     050       2
4560     061       =
Print and store ∂rC in r27
4601     119     print
4602     107     STO MEM
4603     050       2
4604     055       7
4605     109     RCL MEM
4606     050       2
4607     055       7
4608     047       /
Recall h-
4649     109     RCL MEM
4650     057       9
4651     056       8
4652     061       =
Print and store ∂rC/h- in r28
4693     119     print
4694     107     STO MEM
4695     050       2
4696     056       8
4697     040       (
4698     109     RCL MEM
```

```
4699     050       2
4700     052       4
4701     042       x
4702     109     RCL MEM
4703     050       2
4704     054       6
4705     042       x
4706     109     RCL MEM
4707     050       2
4708     056       8
4709     041       )
Print nrA x nrB x nrC
4750     119     print
4751     047       /
Recall symmetry factor
4792     109     RCL MEM
4793     057       9
4794     057       9
4795     042       x
4796     040       (
4797     112       pi
4798     092     Root
4799     050       2
4800     041       )
4801     061       =
4802     110     LOG e
4803     042       x
Recall R
4844     109     RCL MEM
4845     057       9
4846     054       6
4847     061       =
Print and store -Gr/T in r29
4888     119     print
4889     107     STO MEM
4890     050       2
4891     057       9
4892     117     SUM MEM
4893     054       6
4894     049       1
4895     109     RCL MEM
4896     054       6
4897     049       1
Print cumulative -G/T
4938     119     print
4939     040       (
4940     049       1
4941     046       .
4942     053       5
4943     042       x
4944     109     RCL MEM
4945     057       9
4946     054       6
4947     041       )
4948     117     SUM MEM
4949     050       2
4950     057       9
4951     117     SUM MEM
4952     054       6
4953     050       2
4954     109     RCL MEM
4955     054       6
4956     050       2
Print (Ht+Hr)/T
4997     119     print
4998     109     RCL MEM
4999     050       2
5000     057       9
Print Sr non-linear molecules
5041     119     print
5042     043       +
5043     109     RCL MEM
5044     049       1
5045     056       8
5046     061       =
Print St+Sr
5087     119     print
5088     034     Set flag
5089     049       1
5090     096     LABEL
5091     086     V
5092     109     RCL MEM
5093     051       3
5094     049       1
Print wavelength cm^-1
5135     119     print
5136     042       x
5137     109     RCL MEM
5138     057       9
5139     052       4
5140     061       =
Print true frequency v
5181     119     print
5182     042       x
5183     050       2
5184     042       x
5185     112       pi
5186     042       x
5187     109     RCL MEM
5188     057       9
5189     056       8
5190     061       =
5191     119     print
Store hv in r43
5232     107     STO MEM
5233     052       4
5234     051       3
Recall temperature K
5275     109     RCL MEM
5276     048       0
5277     056       8
5278     042       x
5279     109     RCL MEM
5280     057       9
5281     055       7
5282     061       =
Print kT
5323     119     print
Store kT in r44
5364     107     STO MEM
```

```
5365    052     4
5366    052     4
5367    109     RCL MEM
5368    052     4
5369    051     3
5370    047     /
5371    109     RCL MEM
5372    052     4
5373    052     4
5374    061     =
Store hv/kT = x in r45, print
5415    119     print
5416    107     STO MEM
5417    052     4
5418    053     5
Calculate Sv
5459    040     (
5460    109     RCL MEM
5461    057     9
5462    054     6
5463    042     x
5464    109     RCL MEM
5465    052     4
5466    053     5
5467    047     /
5468    040     (
5469    040     (
5470    049     1
5471    064     Inverse
5472    110     LOG e
5473    091     Exponent
5474    109     RCL MEM
5475    052     4
5476    053     5
5477    041     )
5478    045     -
5479    049     1
5480    041     )
5481    061     =
5482    107     STO MEM
5483    052     4
5484    054     6
5485    040     (
5486    049     1
5487    045     -
5488    040     (
5489    040     (
5490    049     1
5491    064     Inverse
5492    110     LOG e
5493    041     )
5494    091     Exponent
5495    109     RCL MEM
5496    052     4
5497    053     5
5498    111     +/-
5499    041     )
5500    041     )
5501    110     LOG e
5502    042     x
5503    109     RCL MEM
5504    057     9
5505    054     6
5506    061     =
5507    107     STO MEM
5508    052     4
5509    055     7
5510    040     (
5511    109     RCL MEM
5512    052     4
5513    054     6
5514    045     -
5515    109     RCL MEM
5516    052     4
5517    055     7
5518    041     )
5519    107     STO MEM
5520    052     4
5521    056     8
Print Sv at w without degeneracy
5562    119     print
5563    042     x
Recall degeneracy D
5604    109     RCL MEM
5605    051     3
5606    053     5
5607    061     =
Print and store total Sv in r49
5648    119     print
5649    117     SUM MEM
5650    052     4
5651    057     9
Sum cumulative -G/T
5692    117     SUM MEM
5693    054     6
5694    049     1
5695    109     RCL MEM
5696    054     6
5697    049     1
Print cumulative -G/T
5738    119     print
5739    109     RCL MEM
5740    052     4
5741    057     9
Print cumulative Sv
5782    119     print
5783    047     /
5784    054     6
5785    046     .
5786    048     0
5787    050     2
5788    050     2
5789    049     1
5790    052     4
5791    049     1
5792    053     5
5793    101     EE
5794    049     1
5795    054     6
5796    061     =
```

```
5797     119     print
ergs per molecule per degree
5838     042       x
5839     109     RCL MEM
5840     048       0
5841     056       8
5842     061       =
Print sT
5883     119     print
5884     117     SUM MEM
5885     053       5
5886     048       0
5887     109     RCL MEM
5888     053       5
5889     048       0
Print svT
5930     119     print
Compute Cv at T with Moore formula
Cv=Rx^2/2(coshx-1)
6011     040       (
6012     040       (
6013     109     RCL MEM
6014     057       9
6015     054       6
6016     042       x
6017     040       (
6018     109     RCL MEM
6019     052       4
6020     053       5
6021     113     Square
6022     041       )
6023     041       )
6024     047       /
6025     040       (
6026     050       2
6027     042       x
6028     040       (
6029     109     RCL MEM
6030     052       4
6031     053       5
6032     104     Hyperbol
6033     099     Cosine
6034     045       -
6035     049       1
6036     041       )
6037     041       )
6038     061       =
Print cumulative Cv
6079     119     print
6080     117     SUM MEM
6081     053       5
6082     049       1
6083     117     SUM MEM
6084     054       6
6085     050       2
6086     109     RCL MEM
6087     054       6
6088     050       2
Print cumulative Cv
6129     119     print
6130     109     RCL MEM
6131     053       5
6132     049       1
Print SumCvv
6173     119     print
6174     047       /
6175     054       6
6176     046       .
6177     048       0
6178     050       2
6179     050       2
6180     049       1
6181     052       4
6182     049       1
6183     053       5
6184     101       EE
6185     049       1
6186     054       6
6187     061       =
Print ergs per molecule per degree
6228     119     print
6229     042       x
6230     109     RCL MEM
6231     048       0
6232     056       8
6233     061       =
Print cvvT
6274     119     print
6275     117     SUM MEM
6276     053       5
6277     050       2
6278     109     RCL MEM
6279     053       5
6280     050       2
Print SumcvvT
6321     119     print
6322     035     If flag
6323     053       5
6324     125      |}|
6325     035     If flag
6326     052       4
6327     123      |{|
6328     035     If flag
6329     051       3
6330     033       !
6331     035     If flag
6332     050       2
6333     099     Cosine
6334     109     RCL MEM
6335     051       3
6336     050       2
6337     107     STO MEM
6338     051       3
6339     049       1
6340     109     RCL MEM
6341     051       3
6342     054       6
6343     107     STO MEM
6344     051       3
6345     053       5
```

```
6346     064     Inverse
6347     034     Set flag
6348     049       1
6349     034     Set flag
6350     050       2
6351     086     V
6352     096     LABEL
6353     099     Cosine
6354     109     RCL MEM
6355     051       3
6356     051       3
6357     107     STO MEM
6358     051       3
6359     049       1
6360     109     RCL MEM
6361     051       3
6362     055       7
6363     107     STO MEM
6364     051       3
6365     053       5
6366     064     Inverse
6367     034     Set flag
6368     050       2
6369     034     Set flag
6370     051       3
6371     086     V
6372     096     LABEL
6373     033       !
6374     109     RCL MEM
6375     051       3
6376     052       4
6377     038     If zero
6378     125      ¦}¦
6379     107     STO MEM
6380     051       3
6381     049       1
6382     109     RCL MEM
6383     051       3
6384     055       7
6385     107     STO MEM
6386     051       3
6387     053       5
6388     064     Inverse
6389     034     Set flag
6390     051       3
6391     034     Set flag
6392     052       4
6393     086     V
6394     096     LABEL
6395     123      ¦{¦
6396     096     LABEL
6397     125      ¦}¦
6398     040       (
6399     109     RCL MEM
6400     052       4
6401     057       9
6402     043       +
6403     109     RCL MEM
6404     053       5
6405     049       1
6406     041       )
Print Sv+Cv
6447     119     print
6448     107     STO MEM
6449     053       5
6450     051       3
6451     040       (
6452     109     RCL MEM
6453     053       5
6454     048       0
6455     043       +
6456     109     RCL MEM
6457     053       5
6458     050       2
6459     041       )
Print sT+cvvT
6500     119     print
6501     107     STO MEM
6502     053       5
6503     052       4
6504     040       (
6505     040       (
6506     040       (
6507     109     RCL MEM
6508     049       1
6509     056       8
6510     043       +
6511     109     RCL MEM
6512     050       2
6513     050       2
6514     043       +
6515     109     RCL MEM
6516     050       2
6517     057       9
6518     041       )
Print St+Sr
6559     119     print
6560     043       +
6561     109     RCL MEM
6562     052       4
6563     057       9
6564     041       )
6565     107     STO MEM
6566     053       5
6567     053       5
Print St+Sr+Sv
6608     119     print
6609     043       +
6610     109     RCL MEM
6611     053       5
6612     049       1
6613     061       =
6614     107     STO MEM
6615     053       5
6616     054       6
Print St+Sr+Sv+Cv
6657     119     print
6658     042       x
6659     109     RCL MEM
6660     048       0
```

```
6661     056       8
6662     061       =
Print [St+Sr+Sv+Sv]T
6703     119     print
6704     107     STO MEM
6705     053       5
6706     055       7
6707     047       /
6708     054       6
6709     046       .
6710     048       0
6711     050       2
6712     050       2
6713     049       1
6714     052       4
6715     049       1
6716     053       5
6717     101       EE
6718     049       1
6719     054       6
6720     061       =
6721     119     print
[st+sr+sv+cv]T printed
6762     040       (
6763     109     RCL MEM
6764     053       5
6765     053       5
6766     042       x
6767     109     RCL MEM
6768     048       0
6769     056       8
6770     041       )
Print [St+Sr+Sv]T
6811     119     print
6812     047       /
6813     054       6
6814     046       .
6815     048       0
6816     050       2
6817     050       2
6818     049       1
6819     052       4
6820     049       1
6821     053       5
6822     101       EE
6823     049       1
6824     054       6
6825     061       =
Print [st+sr+sv]T
6866     119     print
6867     109     RCL MEM
6868     054       6
6869     049       1
Print cumulative -G/T
6910     119     print
6911     042       x
6912     109     RCL MEM
6913     048       0
6914     056       8
6915     061       =
Print -G
6956     119     print
6957     109     RCL MEM
6958     054       6
6959     050       2
Print cumulative H/T
7000     119     print
7001     042       x
7002     109     RCL MEM
7003     048       0
7004     056       8
7005     061       =
Print cumulative H
7046     119     print
7047     048       0
7048     107     STO MEM
7049     053       5
7050     054       6
7051     107     STO MEM
7052     053       5
7053     053       5
7054     107     STO MEM
7055     053       5
7056     052       4
7057     107     STO MEM
7058     053       5
7059     051       3
7060     107     STO MEM
7061     053       5
7062     050       2
7063     107     STO MEM
7064     053       5
7065     049       1
7066     107     STO MEM
7067     053       5
7068     048       0
7069     107     STO MEM
7070     052       4
7071     057       9
7072     107     STO MEM
7073     049       1
7074     056       8
7075     096     LABEL
7076     115     Sine
7077     044     Reset
7078     002     HALT
```
